# Improved circuit implementation of the HHL algorithm and its simulations on QISKIT

**DOI:** 10.1038/s41598-022-17660-8

**Published:** 2022-08-02

**Authors:** Meng Zhang, Lihua Dong, Yong Zeng, Ning Cao

**Affiliations:** 1 School of Communication Engineering, Xidian University, Xi’an, 710071 China; 2 School of Network and Information Security, Xidian University, Xi’an, 710071 China

**Keywords:** Mathematics and computing, Information technology

## Abstract

In 2019, Yonghae Lee et al. combined the circuit implementation of the Harrow–Hassidim–Lloyd (HHL) algorithm with a classical computer, and designed a hybrid HHL algorithm to reduce experimental errors caused by decoherence and so on. However, the improvement is achieved only in the auxiliary quantum coding phase, and no quantum resource reduction is done on the quantum phase estimation and inverse quantum phase estimation stages. At the same time, the circuit improvement illustration on a $2\times 2$ linear system just has the result and no specific process. In this paper, based on the idea of the hybrid HHL algorithm and a generic circuit of HHL algorithm, an improved circuit implementation of the HHL algorithm is proposed. The feasibility of the improved circuit implementation of the HHL algorithm is verified by IBM's qiskit. The improved circuit illustrations on a $4\times 4$ linear system show that the improved circuit implementation of the HHL algorithm can effectively reduce quantum resources without losing the fidelity of the results. Thus the improved circuit implementation of the HHL algorithm can further avoid some result errors than the existing implementation methods.

## Introduction

Quantum computing is an operation method that follows the operating laws of quantum mechanics. Compared with traditional classical computing, quantum computing achieves an exponential speedup on some problems. For example, the Shor quantum algorithm^1^ is famous for factoring large integers in polynomial time. Grover algorithm^2^ achieves an exponential speedup in searching data. While the HHL algorithm^3^ for solving linear systems of equations with exponential speedup over the best known classical algorithms.

However, during the operation of quantum circuits, due to the limitations of current technology, the errors of quantum gates, experimental errors and decoherence will introduce errors in the experimental process^4–6^. Therefore, it is necessary to reduce the number of gates and the overall running time of the algorithm. In view of this, the realization and improvement of quantum circuits has attracted the attention of various fields.

Among them, in terms of quantum circuit implementation and improvement of cryptographic algorithms, Langenberg et al. proposed the quantum circuit implementation and improvement of AES cryptographic algorithm^7^, and many scholars have made further improvements on it^8,9^.

In circuit synthesis of quantum algorithms, Monz et al. implemented the use of Shor's algorithm to factorize numbers 15^10^ using 7 qubits and 4 cache qubits with efficient control. Diao et al. proposed the quantum circuit composition of Grover algorithm^11^. Markus Grassl successfully used the Grover algorithm to realize the exhaustive key search for AES^12^. The quantum circuit implementation and improvement of the HHL algorithm also attracted a lot of attention. Among them, by introducing the variable time amplitude amplification algorithm into the HHL algorithm, Ambainis et al. reduced the number of repeated runs required to obtain the correct answer, thereby reducing the running time of the algorithm^13^, but did not give a specific quantum circuit implementation. Yudong Cao et al. proposed a generic circuit of HHL algorithm^14^. Yonghae Lee et al. gave a hybrid HHL quantum algorithm^6^ by combining with classical computers, which effectively reduces the quantum gate resources used in the auxiliary qubit rotation part of the HHL algorithm. Compared with the generic circuit of HHL algorithm proposed by Cao et al., the hybrid HHL algorithm uses smaller quantum resources in the auxiliary qubit rotation stage. However, the improvement has only limited to the auxiliary quantum encoding stage, and no quantum resource can be reduced on the quantum phase estimation and inverse quantum phase estimation stages. Meanwhile, in the circuit improvement verification, there is only improvement result on a $2\times 2$ linear system and no specific realization process is given.

In view of this, inspired by the idea of combining with classical computers and the generic circuit of HHL algorithm, we provide an improved circuit implementation of the HHL algorithm to further reducing the number of quantum gates, thus further avoiding some result errors caused by quantum gate errors. The experimental results show that our improved circuit implementation of the HHL algorithm effectively reduces the consumption of quantum resources without losing the fidelity of the results.

### Basic definitions

- *k-fixed*^6^: Suppose $\lambda_{j} ,\;j = 1, \ldots ,l,$ is all non-zero eigenvalues of the Hermitian matrix *A*, ${b}_{k}^{j}$ is the *k*th bit of the binary representation of the eigenvalues ${\lambda }_{j}$, *j* = 1, …, *l*, defined ${\overline{m} }_{k}$, *k* $\in N$, as follows
  1 $${\overline{m} }_{k}=\frac{1}{l}\left(\sum _{j=1}^{l}{b}_{k}^{j}\right)$$
  Hermitian matrix *A* is said to be *k-fixed* if it is ${\overline{m} }_{k}$ fixed at 0 or 1.
- *n-estimated*^6^: Denoted $\lambda$ as the eigenvalue of the matrix, if it can be represented $\lambda$ by no more than n binary number, it is called *n-estimated.*
- *fidelity f*^15^: In the experiment in this paper, fidelity can be understood as the inner product of two vectors:
  2 $$f=({x}_{theory},{x}_{simulation})$$
  where ${x}_{theory}$ and ${x}_{simulation}$ are normalized values.

### HHL algorithm

The definition of general linear equations satisfies the following conditions:

$$A\overrightarrow{x}=\overrightarrow{b}$$

where **A** is m × n matrix:

The above mentioned is a system of linear equations in the classical algorithm, which is expressed in quantum computation as follows:

$$A\left|x\right.\rangle =\left|b\right.\rangle$$

where $\left|x\right.\rangle$ and $\left|b\right.\rangle$ are quantum states.

The HHL algorithm is used to solve quantum equations. However, when using HHL algorithm to solve the problem, some specific conditions still need to be met. Matrix **A** must be an *n* × *n* square matrix, and **A** must be a Hermitian operator. If not, **A** needs to be transformed into a Hermitian matrix in some way. The core idea of HHL algorithm includes phase estimation, controlled rotation and inverse phase estimation.

The general steps of the HHL algorithm are:

1. First, initialize the quantum state $\left|b\right.\rangle$.
2. The quantum phase estimation method is used to decompose $\left|b\right.\rangle$, into the superposition of the linear combination of eigenvectors of **A**.
3. After inverting the matrix **A**, we get the quantum state ${A}^{-1}\left|x\right.\rangle =\left|b\right.\rangle$.
4. Canceling the eigenvalues stored in the register by using the inverse quantum phase estimation method.

#### Quantum circuit implementation of HHL algorithm

The quantum circuit implementation of the HHL algorithm requires the use of three quantum registers, denoted as $Ancilla$, $\mathit{Re}g.C$ and $\mathit{Re}g.B$, where

- $Ancilla$ is used to store auxiliary qubits;
- $\mathit{Re}g.C$ is used to store the binary representation of the eigenvalues of the coefficient matrix A;
- $\mathit{Re}g.B$ is used to store the vector solution of a system of linear equations when the measurement of the contents of the $Ancilla$ quantum register is 1.
- Initially, all three quantum registers are set to $\left|0\right.\rangle$ state.

**Figure 1:**
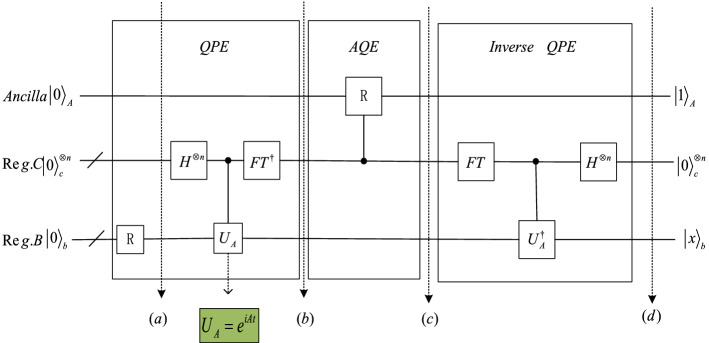
Circuit overview diagram of HHL algorithm.

As shown in Fig. 1, the quantum circuit implementation process of the HHL algorithm is mainly composed of three stages, namely: quantum phase estimation (Quantum Phase Estimation, QPE), auxiliary quantum encoding (Ancilla quantum encoding, AQE) and inverse quantum phase estimation (Inverse Quantum Phase Estimation, Inverse QPE) of which

- QPE, that is, Quantum Phase Estimation. In the HHL algorithm, if the quantum register is measured after the QPE stage, then the binary representation of the eigenvalues of the linear equation system will be obtained, denoted as $\left|{\theta }_{0}{\theta }_{1}...{\theta }_{n-1}\right.\rangle$.
  Firstly, in the preparation phase, the first *n* qubits are initialized to 0, and the last *m* qubits are initialized to the quantum state $\left|u\right.\rangle$, so the initial state of the register is ${\left|0\right.\rangle }^{\otimes n}\left|u\right.\rangle$, then apply the H gate to n qubits.
  3 $$\begin{aligned}\left|{\varphi }_{1}\right.\rangle &=\left({H}^{\otimes n}\otimes I\right){\left|0\right.\rangle }^{\otimes n}\left|u\right.\rangle\\ &=\frac{1}{\sqrt{{2}^{n}}}\sum _{{x}_{0}{x}_{1}\dots {x}_{n-1}\in \{0,1{\}}^{n}}\left|{x}_{0}{x}_{1}\dots {x}_{n-1}\right.\rangle \left|u\right.\rangle ,\,\,\,{x}_{i}\in \{0,1\}\end{aligned}$$
  Secondly, using n controlled *U* gates, add the phase ${e}^{2\pi i\theta }$ to the probability amplitude.
  4 $$\begin{aligned} \left| {\varphi_{2} } \right.\rangle & = \frac{1}{{2^{\frac{n}{2}} }}\underbrace {{\left| 0 \right.\rangle + e^{{2\pi i2^{n - 1} \left( {0.\theta_{0} \theta_{2} ,...,\theta_{n - 1} } \right)}} \left| 1 \right.\rangle }}_{{1{\text{st}}\;qubit}} \otimes ... \otimes \underbrace {{\left| 0 \right.\rangle + e^{{2\pi i2^{1} \left( {0.\theta_{0} \theta_{1} ,...,\theta_{n - 1} } \right)}} \left| 1 \right.\rangle }}_{{n - 1{\text{st}}\;qubit}} \\ &\quad \otimes \underbrace {{\left| 0 \right.\rangle + e^{{2\pi i2^{0} \left( {0.\theta_{0} \theta_{1} ,...,\theta_{n - 1} } \right)}} \left| 1 \right.\rangle }}_{{n{\text{st}}\;qubit}}\left| u \right.\rangle \\ \end{aligned}$$
  Finally, the inverse QFT (quantum Fourier transform) is performed on the $\left|{\varphi }_{2}\right.\rangle$ quantum state. This step extracts the target value $\theta$ from the probability amplitude to the ground state. For qubits $\left|{x}_{k}\right.\rangle ,k=\mathrm{1,2},\dots n.$
  5 $$\left|{x}_{k}\right.\rangle =\frac{1}{\sqrt{2}}\left(\left|0\right.\rangle +{\left(-1\right)}^{{\theta }_{k}}{e}^{\frac{1}{2}\pi i{\theta }_{k+1}}...{e}^{\frac{1}{{2}^{n-1}}\pi i{\theta }_{n-1}}\left|1\right.\rangle \right).$$
  To extract ${\theta }_{k}$ into the quantum state, it is necessary to perform phase rotation on the qubit $\left|{x}_{k}\right.\rangle$ before applying the H gate to the qubit $\left|{x}_{k}\right.\rangle$, and the phase selection angle is ${e}^{\frac{1}{2}\pi i{\theta }_{k+1}}...{e}^{\frac{1}{{2}^{n-1}}\pi i{\theta }_{n-1}}$.
  6 $$H\otimes CU({e}^{\frac{1}{2}\pi i{\theta }_{k+1}}...{e}^{\frac{1}{{2}^{n-1}}\pi i{\theta }_{n-1}})\left|{\theta }_{0}\right.\rangle \left|{x}_{k}\right.\rangle =\left|{\theta }_{0}\right.\rangle \left|{\theta }_{k}\right.\rangle$$
  Extract all $\left|{\theta }_{0}{\theta }_{1}...{\theta }_{n-1}\right.\rangle$ into qubits to get the binary representation of the eigenvalues of the matrix
  7 $$\left|{\varphi }_{3}\right.\rangle =\left|{\theta }_{0}{\theta }_{1}...{\theta }_{n-1}\right.\rangle \left|u\right.\rangle$$
  Measure the result to get $\left|{\theta }_{0}{\theta }_{1}...{\theta }_{n-1}\right.\rangle$, which is the binary representation of the eigenvalues of the desired matrix.
  In the entire HHL algorithm, the unitary operator $U={U}_{A}={e}^{iAt}={\sum }_{j=0}^{N-1}{e}^{i{\lambda }_{j}t}\left|{u}_{j}\right.\rangle \left.\langle {u}_{j}\right|$, $\left|b\right.\rangle ={\sum }_{j=0}^{N-1}{b}_{j}\left|{u}_{j}\right.\rangle$, where *t* is a constant that can be set by itself. Therefore, in the entire HHL algorithm, the state of time, that is, the moment in Fig. 1 is $\left(b\right)$:
  8 $$\begin{aligned} \left| 0 \right.\rangle_{A} & \otimes \mathop \sum \limits_{j = 0}^{N - 1} b_{j} \left| {\lambda_{j} } \right.\rangle_{nl} \otimes \left| {u_{j} } \right.\rangle \\ \left| u \right.\rangle & = \mathop \sum \limits_{j = 0}^{N - 1} b_{j} \left| {u_{j} } \right.\rangle ,\quad \left| {\lambda_{j} } \right.\rangle_{nl} = \frac{1}{{\sqrt {2^{n} } }}\mathop \sum \limits_{i = 0}^{n - 1} \left| {\theta_{i} } \right.\rangle \\ \end{aligned}$$
  Here ${\left|{\lambda }_{j}\right.\rangle }_{nl}$ is the n-bit binary representation of “${\lambda }_{j}$”.
- In the AQE stage, a controlled rotation operation on auxiliary qubits is performed as follows:
  9 $${\left|0\right.\rangle }_{A}\otimes {\left|{\lambda }_{j}\right.\rangle }_{nl}\to \left(\sqrt{1-\frac{{c}^{2}}{{\lambda }_{j}^{2}}}{\left|0\right.\rangle }_{A}+\frac{c}{{\lambda }_{j}}{\left|1\right.\rangle }_{A}\right)\otimes {\left|{\lambda }_{j}\right.\rangle }_{nl}$$
  Here $c$ is a normalizing constant.
  After the AQE part, the state of the system at moment Fig. 1c is:
  10 $$\sum _{j=0}^{N-1}{b}_{j}{\left|{\lambda }_{j}\right.\rangle }_{nl}\left|{u}_{j}\right.\rangle \left(\sqrt{1-\frac{{c}^{2}}{{\lambda }_{j}^{2}}}{\left|0\right.\rangle }_{A}+\frac{c}{{\lambda }_{j}}{\left|1\right.\rangle }_{A}\right)$$
- Inverse QPE stage is the inverse operation of the QPE stage. After the Inverse QPE stage, the $\left|{\lambda }_{j}\right.\rangle$ in the superposition state in the $\mathit{Re}g.C$ register will become $\left|0\right.\rangle$, at this time, the state of the entire quantum system is Fig. 1$(d)$:
  11 $$\sum _{j=0}^{N-1}{b}_{j}{\left|0\right.\rangle }_{nl}\left|{u}_{j}\right.\rangle \left(\sqrt{1-\frac{{c}^{2}}{{\lambda }_{j}^{2}}}{\left|0\right.\rangle }_{A}+\frac{c}{{\lambda }_{j}}{\left|1\right.\rangle }_{A}\right)$$
  After the above three stages are completed, the status in the $\mathit{Re}g.C$ register changes to the ${\left|0\right.\rangle }^{\otimes n}$ state, and the auxiliary qubit is measured on the Z-axis. If the outcome is 1, the register is in the post-measurement state:
  12 $$\left|x\right.\rangle =\left(\sqrt{\frac{1}{{\sum }_{j=0}^{N-1}{\left|{b}_{j}\right|}^{2}/{\left|{\lambda }_{j}\right|}^{2}}}\right)\sum _{j=0}^{N-1}\frac{{b}_{j}}{{\lambda }_{j}}{\left|0\right.\rangle }_{nl}\left|{u}_{j}\right.\rangle .$$
  which up to a normalisation factor corresponds to the solution.

#### Generic circuit of HHL algorithm

**Figure 2:**
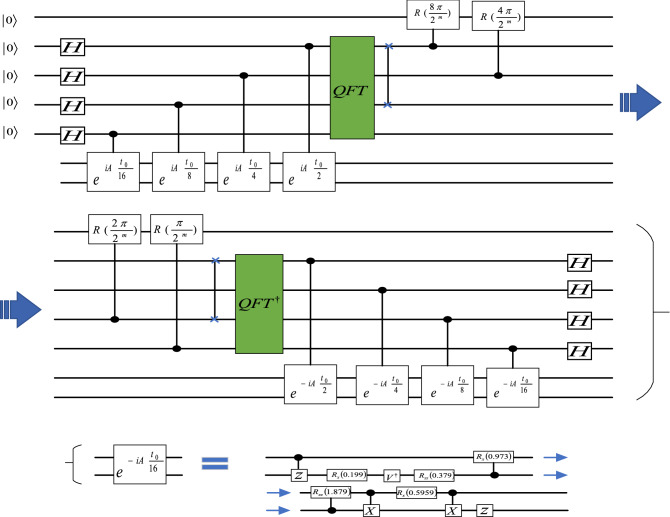
Generic circuit of HHL algorithm.

In 2012, Yudong Cao et al. proposed an efficient and generic circuit of HHL algorithm^14^. In this design, the Group Leader Optimization Algorithm was used to find the circuit decomposition of the Hamiltonian analog operator $\mathrm{exp}\left[\mathrm{iA}\left(2\uppi /16\right)\right]$^16,17^. Then simply multiply the offset angles of all the revolving gates in the circuit by a factor of 2, 4, and 8 to get the operators $\mathit{exp}\left[iA\left(2\pi /8\right)\right]$, $\mathit{exp}\left[iA\left(2\pi /4\right)\right]$ and $\mathit{exp}\left[iA\left(2\pi /2\right)\right]$. They show a $4\times 4$ linear system as shown in Fig. 2 below.

#### Hybrid HHL algorithm

Yonghae Lee et al. proposed a hybrid HHL algorithm in 2019^6^. The implementation of the algorithm is based on the following two characteristics given in the paper:

##### **Characteristic 1.**

The fidelity of HHL algorithm results can reach 1 only when all eigenvalues of the matrix can perfectly *n-estimated*.

For example, for a $2\times 2$ matrix, at that time $\lambda_{n = 2} = 1,2\;{\text{or}}\;3$, its eigenvalues are *2-estimated*. Using 2-bit quantum registers, its fidelity can reach 1, but if its eigenvalues exceed 3 or are decimals, it is not perfectly *2-estimated*, so the fidelity cannot reach 1 when using 2-bit quantum registers.

##### **Characteristic 2.**

 If the eigenvalues of the matrix are not perfectly *n-estimated*, then the fidelity of the algorithm's results will be positively related to the number of extra quantum registers used.

This is because for matrices with non-perfect *n-estimated* eigenvalues, additional qubit registers are needed to represent the eigenvalues of the matrix and to control the phase rotation. In other words, additional quantum are required to improve the fidelity of the results. For example, the eigenvalues of a $2\times 2$ matrix ${\lambda }_{n=2}=4$ are not perfectly represented by *2-estimated*. In this case, in order to make the fidelity close to 1, it is necessary to use 3 qubits to store the eigenvalue information of the matrix.

From Characteristic 1 and Characteristic 2 we note that once the eigenvalues of the matrix are not perfectly *n-estimated*, the number of qubits in quantum registers that need to be used to achieve high fidelity increases dramatically.When all eigenvalues can be perfectly *n-estimated*, *n—*scale quantum registers are also required to ensure high fidelity. Therefore, in the implementation process of the HHL algorithm, in order to reduce the circuit complexity, it is necessary to choose a matrix with perfect *n-estimated* as much as possible. At the same time, the literature^6^ pointed out the following conclusion.

#### Conclusion

In the execution of the HHL algorithm, for a perfect *n-estimated* matrix, if it has *k-fixed* eigenvalues, it can be implemented with a smaller depth quantum circuit, and compared with the original HHL algorithm, the circuit complexity is reduced, and the smaller depth quantum circuit of HHL algorithm has higher fidelity.

**Figure 3:**
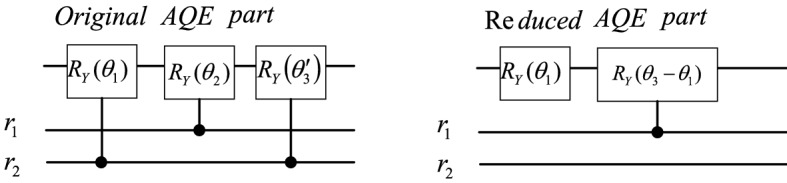
The quantum circuits before and after the improvement of the AQE stage in the hybrid HHL algorithm.

Reference^6^ uses the above conclusion to improve the quantum circuit of the AQE stage of the HHL algorithm, and gives the improved quantum circuit of the $2\times 2$ matrix as shown in Fig. 3. In the hybrid HHL algorithm, the feedforward combined with the information obtained by classical calculation after quantum phase estimation effectively reduces the number of quantum gates of the original HHL algorithm.

However, the implementation details of the improvement of the AQE stage of the HHL algorithm are not described in this algorithm, and the quantum circuits in the QPE and inverse QPE stages are not improved.

This paper will take the generic circuit of HHL algorithm proposed by Yudong Cao et al. as the framework, and use the design idea of the hybrid HHL algorithm proposed by Yonghae Lee et al. to improve the implementation circuit of the HHL algorithm as a whole.

## Result

### Preparation before implementation

*QPE is repeatedly performed to obtain the information of the eigenvalues*: in the quantum phase estimation part, the binary representation of the eigenvalues has been stored in $\mathit{Re}g.B$ each qubit of the quantum register after the phase estimation. If the $\mathit{Re}g.B$ quantum register is measured on the Z-axis, then the $\mathit{Re}g.B$ quantum register can be collapsed to an eigenvalue of the matrix, and the measurement process can be repeated^6^.

*Secondly, the prior information is obtained by combining probability statistics with classical computers*: whether the information of the matrix eigenvalues obtained in the QPE stage is statistically observed to be *k-fixed.* If the *k*-*fixed* characteristic is not observed in the statistical eigenvalue information, more qubit registers need to be used to store the matrix eigenvalues in order to discover the *k-fixed* characteristic.

### Simplification of quantum circuits

In this subsection, according to the *k-fixed* property of the matrix, we give a specific implementation method to reduce the number of quantum gates required to realize the HHL quantum circuit, and compare it with the generic circuit of HHL algorithm.

1. Quantum phase estimation stage
  When the eigenvalue of the matrix, that is, $\lambda$ is k-fixed, the kth binary value ${\overline{m} }_{k}$ of all eigenvalues of the matrix is either 0 or 1.
  Firstly, when ${\overline{m} }_{k}=0$, in the first part of the QPE, n − 1 *H* gates are used to add to the qubit except the kth qubit.
  13 $$\left|{\varphi }_{1}\right.\rangle =\left({H}^{\otimes n-1}\otimes I\right){\left|0\right.\rangle }^{\otimes n}\left|u\right.\rangle =\frac{1}{\sqrt{{2}^{n}}}\sum _{{x}_{0}{x}_{1}...{x}_{n-1}\in {\left\{0,1\right\}}^{n}}\left|0\right.\rangle \left|{x}_{0}{x}_{1}...{x}_{k-1}{x}_{k+1}...{x}_{n}\right.\rangle \left|u\right.\rangle$$
  According to our definition of ${\overline{m} }_{k}$, the state of Eq. (13) is the same as the state of Eq. (3) at this time.
  Secondly, when using n − 1 controlled gate to add phase ${e}^{2\pi i\theta }$ to the probability amplitude, it does not have to act on the kth qubit.
  14 $$\begin{aligned} \left| {\varphi_{2} } \right.\rangle & = \frac{1}{{2^{\frac{n}{2}} }}\underbrace {{\left| 0 \right.\rangle + e^{{2\pi i2^{n - 1} \left( {0.\theta_{0} \theta_{1} ,...,\theta_{n - 1} } \right)}} \left| 1 \right.\rangle }}_{{1{\text{st}}\;qubit}} \otimes ...\underbrace {\left| 0 \right.\rangle }_{{k{\text{st}}\;qubit}}... \otimes \underbrace {{\left| 0 \right.\rangle + e^{{2\pi i2^{1} \left( {0.\theta_{0} \theta_{1} ,...,\theta_{n - 1} } \right)}} \left| 1 \right.\rangle }}_{{n - 1{\text{st}}\;qubit}} \\ & \quad \otimes \underbrace {{\left| 0 \right.\rangle + e^{{2\pi i2^{0} \left( {0.\theta_{0} \theta_{1} ,...,\theta_{n - 1} } \right)}} \left| 1 \right.\rangle }}_{{n{\text{st}}\;qubit}}\left| u \right.\rangle \\ \end{aligned}$$
  The state of Eq. (14) is the same as the state of Eq. (4) at this time.
  Finally, $\left|{\varphi }_{2}\right.\rangle$ quantum state performs inverse quantum Fourier transform, which extracts the target value $\theta$ from the probability amplitude into the ground state.
  For qubits $\left|{x}_{k}\right.\rangle , k=\mathrm{1,2},...n$. We already know that the matrix is *k-fixed* and ${\overline{m} }_{k}=0$, so there is no need to adjust $\left|{x}_{k}\right.\rangle$ perform phase rotation and directly measure $\left|{x}_{k}\right.\rangle$, we can get $\left|0\right.\rangle$.
  15 $$\left|{\varphi }_{3}\right.\rangle =\left|{\theta }_{0}{\theta }_{1}...{\theta }_{k-1}0{\theta }_{k+1}...{\theta }_{n-1}\right.\rangle \left|u\right.\rangle$$
  The state of Eq. (15) is also the same as the state of Eq. (7) at this time.
  When the matrix is *k-fixed* and ${\overline{m} }_{k}=0$. According to the definition of ${\overline{m} }_{k}$, the QPE part of the improved circuit implementation of the HHL algorithm is the same as the QPE part of the original HHL algorithm. For ${\overline{m} }_{k}=1$, it is similar to the process of ${\overline{m} }_{k}=0$, except that in the first stage of QPE, the initialization state of the kth qubit is changed to $\left|1\right.\rangle .$
  For example, when the eigenvalue of a perfect *4-estimated* matrix is ${\lambda }_{j}$ *2-fixed*, one can get
  16 $$\begin{aligned}{\left|\varphi \right.\rangle }_{{\theta }_{1}=0}&=\frac{\left(\left|0\right.\rangle +{e}^{2\pi i\left(\frac{1}{2}{\theta }_{3}\right)}\left|1\right.\rangle \right)\left(\left|0\right.\rangle +{e}^{2\pi i\left(\frac{1}{2}{\theta }_{2}+\frac{1}{4}{\theta }_{3}\right)}\left|1\right.\rangle \right)}{4}\\ &\quad\cdot \frac{\left(\left|0\right.\rangle +{e}^{2\pi i\left(\frac{1}{4}{\theta }_{2}+\frac{1}{8}{\theta }_{3}\right)}\left|1\right.\rangle \right)\left(\left|0\right.\rangle +{e}^{2\pi i\left(\frac{1}{2}{\theta }_{0}+\frac{1}{8}{\theta }_{2}+\frac{1}{16}{\theta }_{3}\right)}\left|1\right.\rangle \right)}{4}\left|\mu \right.\rangle \to \left|{\theta }_{0}{\theta }_{2}{\theta }_{3}\right.\rangle \left|0\right.\rangle \left|\mu \right.\rangle .\end{aligned}$$
  17 $$\begin{aligned}{\left|\varphi \right.\rangle }_{{\theta }_{1}=1}&=\frac{\left(\left|0\right.\rangle +{e}^{2\pi i\left(\frac{1}{2}{\theta }_{3}\right)}\left|1\right.\rangle \right)\left(\left|0\right.\rangle +{e}^{2\pi i\left(\frac{1}{2}{\theta }_{2}+\frac{1}{4}{\theta }_{3}\right)}\left|1\right.\rangle \right)}{4}\\ & \quad\cdot \frac{\left(\left|0\right.\rangle +{e}^{2\pi i\left(\frac{1}{4}{\theta }_{2}+\frac{1}{8}{\theta }_{3}\right)}\left|1\right.\rangle \right)\left(\left|0\right.\rangle +{e}^{2\pi i\left(\frac{1}{2}{\theta }_{0}+\frac{1}{8}{\theta }_{2}+\frac{1}{16}{\theta }_{3}\right)}\left|1\right.\rangle \right)}{4}\left|\mu \right.\rangle \to \left|{\theta }_{0}{\theta }_{2}{\theta }_{3}\right.\rangle \left|1\right.\rangle \left|\mu \right.\rangle .\end{aligned}$$
  According to the above formula, the comparison diagram shown in Fig. 4 can be obtained.

**Figure 4:**
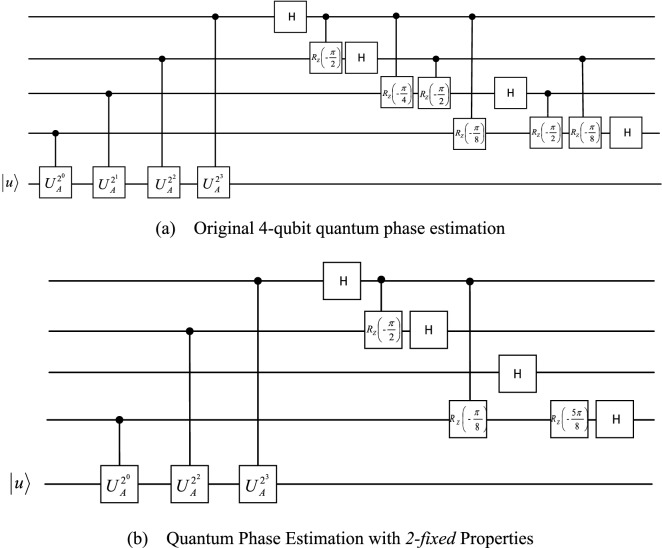
Comparison of quantum phase estimation with and without *4-estimated* and *2-fixed* properties.
  Thus, if the eigenvalues of a perfect *n-estimated* matrix satisfy *k-fixed*. For a qubit representing ${\lambda }_{k}$ in quantum register $\mathit{Re}g.C$, the applied quantum gate can be roughly reduced by a factor of $\frac{n-1}{n}$.
2. Auxiliary quantum encoding phase
  After phase estimation, quantum register $\mathit{Re}g.C$ stores a series of binary superposition states of eigenvalues. The control rotation part is to control the auxiliary qubit according to the superposition state in the quantum register at this time, as shown in Fig. 5 below. Figure 5a is the quantum circuit under the generic circuit of HHL algorithm. Figure 5b is *2-fixed* and $\overline{m}_{1} = 0$ improved quantum circuit implementation. Figure 5c is the improved quantum circuit implementation of 2-fixed and $\overline{m}_{1} = 1$.

**Figure 5:**
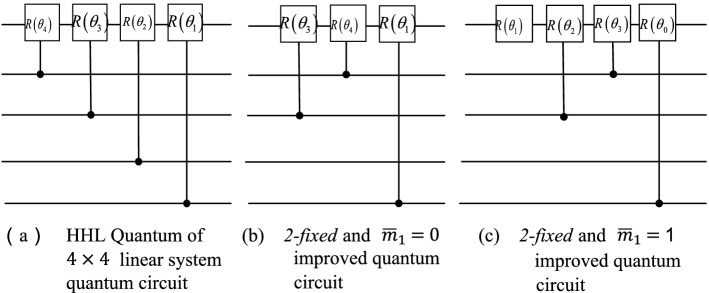
AQE part of the original circuit and the improved circuit.
  After the AQE section, we can get the system status as follows:
  18 $${\left|0\right.\rangle }_{A}\left|{\theta }_{0}{\theta }_{2}{\theta }_{3}\right.\rangle \left|1\right.\rangle \left|\mu \right.\rangle \to \left(\left(\sqrt{1-{\left(\frac{c}{{\lambda }_{{\theta }_{1}=1}}\right)}^{2}}\right){\left|0\right.\rangle }_{A}+\frac{c}{{\lambda }_{{\theta }_{1}=1}}{\left|1\right.\rangle }_{A}\right)\left|{\theta }_{0}{\theta }_{2}{\theta }_{3}\right.\rangle \left|1\right.\rangle \left|\mu \right.\rangle .$$
  19 $${\left|0\right.\rangle }_{A}\left|{\theta }_{0}{\theta }_{2}{\theta }_{3}\right.\rangle \left|0\right.\rangle \left|\mu \right.\rangle \to \left(\left(\sqrt{1-{\left(\frac{c}{{\lambda }_{{\theta }_{1}=0}}\right)}^{2}}\right){\left|0\right.\rangle }_{A}+\frac{c}{{\lambda }_{{\theta }_{1}=0}}{\left|1\right.\rangle }_{A}\right)\left|{\theta }_{0}{\theta }_{2}{\theta }_{3}\right.\rangle \left|0\right.\rangle \left|\mu \right.\rangle .$$
3. Inverse quantum phase estimation
  After the AQE part, the inverse quantum phase estimation and quantum phase estimation have the same simplified circuit implementation, and will not be repeated here. The applied quantum gates can likewise be roughly reduced to the original $\frac{n-1}{n}$.

#### Quantum circuit implementation example

IBM Q provides a Qiskit library based on the Python programming environment that can be used for remote access or emulation with classics. In this section, the Qiskit library is used to simulate a linear system $Ax=b$, where $A$ is a randomly chosen 4 × 4 matrix that is 4-estimated and 1-fixed, $$A = \left[ {\begin{array}{*{20}c} {11} & 5 & { - 1} & { - 1} \\ 5 & {11} & 1 & 1 \\ { - 1} & 1 & {11} & { - 5} \\ { - 1} & 1 & { - 5} & {11} \\ \end{array} } \right]$$, $b={\left(0,0,0,1\right)}^{T}$, with the generic circuit of HHL algorithm and the improved circuit implementation of the HHL algorithm. To obtain the result we run the simulation 1024 times. Here the eigenvalues of matrix A can be accurately stored using four qubits.

**Figure 6:**
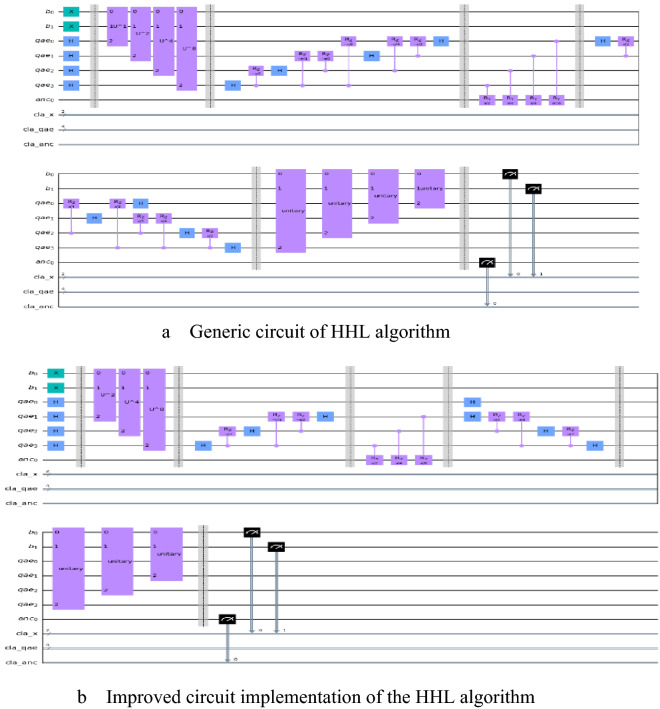
Comparison of the generic circuit of HHL algorithm and improved circuit implementation of the HHL algorithm.

The quantum circuit is shown in Fig. 6 below.

It can be seen from Fig. 6 that the improved circuit implementation of the HHL algorithm uses less quantum gates than the generic circuit of HHL algorithm. On the other hand, the quantum resources and fidelity used to obtain the simulation solution using the generic circuit of HHL algorithm and the simulation solution obtained through the improved circuit implementation of the HHL algorithm are compared as shown in Table 1 below.

**Table 1:** Comparison of simulative and theoretical solutions of $4\times 4$ linear system.

Algorithm	Solution	Fidelity	Depth	Width	Total quantum gate
Theoretical solution	$$\left(\begin{array}{c}\begin{array}{c}\sqrt{\text{0.}{0455}}\\ \sqrt{\text{0.}{0455}}\end{array}\\ \begin{array}{c}\sqrt{\text{0.}{1818}}\\ \sqrt{\text{0.}{7272}}\end{array}\end{array}\right)$$	1	–	–	–
Generic circuit	$$\left(\begin{array}{c}\begin{array}{c}\sqrt{\text{0.}{0412}}\\ \sqrt{\text{0.}{0450}}\end{array}\\ \begin{array}{c}\sqrt{\text{0.}{2450}}\\ \sqrt{\text{0.}{6687}}\end{array}\end{array}\right)$$	0.993	28	14	39
Improved circuit	$$\left(\begin{array}{c}\begin{array}{c}\sqrt{\text{0.}{0371}}\\ \sqrt{\text{0.}{0358}}\end{array}\\ \begin{array}{c}\sqrt{\text{0.}{1687}}\\ \sqrt{\text{0.}{7583}}\end{array}\end{array}\right)$$	0.998	21	14	28

When using quantum circuits to implement the HHL algorithm to solve the linear equation system, the fidelity of the experimental solution cannot reach 1 due to current technical limitations.

Experimental results show that when using improved circuit to solve linear equations, the fidelity of the experimental solution is higher than that of the original HHL general purpose quantum circuit, and it uses less quantum resources.

## Discussion

In general, for linear systems, if the prior condition is satisfied, that is, the eigenvalues of the matrix of the linear equation system have *k-fixed* characteristics, the quantum resources consumption can be reduced without reducing the fidelity of the experimental results. For an n-dimensional linear equation system, in the case of satisfying *k-fixed*, the quantum gate applied to the qubit representing ${\lambda }_{k}$ in the quantum register $\mathit{Re}g.C$ can be roughly reduced to the original $\frac{n-1}{n}$. If we observe more *k-fixed* information in the first step, and record the number of *k-fixed* as ${k}_{num}$, $0<{k}_{num}<=n$, the applied quantum gates can be roughly reduced to the original $\frac{n-{k}_{num}}{n}$, and the reduced circuit depth of the quantum circuit is about 2n.

## Methods

Improved circuit implementation of the HHL algorithm is the same as the generic circuit of HHL algorithm in the initialization part, the difference is that the improved circuit implementation of the HHL algorithm uses the generic circuit of HHL algorithm to achieve the QPE part, and further uses the measured $\mathit{Re}g.B$ quantum register. The information of some matrix eigenvalues is used to assist the construction of the following circuit.

1. Generic circuit of HHL algorithm
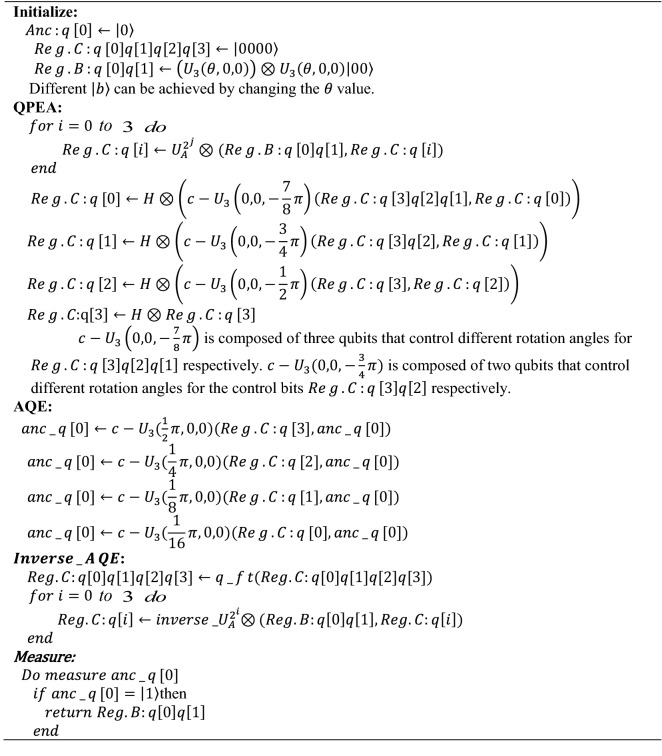
2. Improved circuit implementation of the HHL algorithm
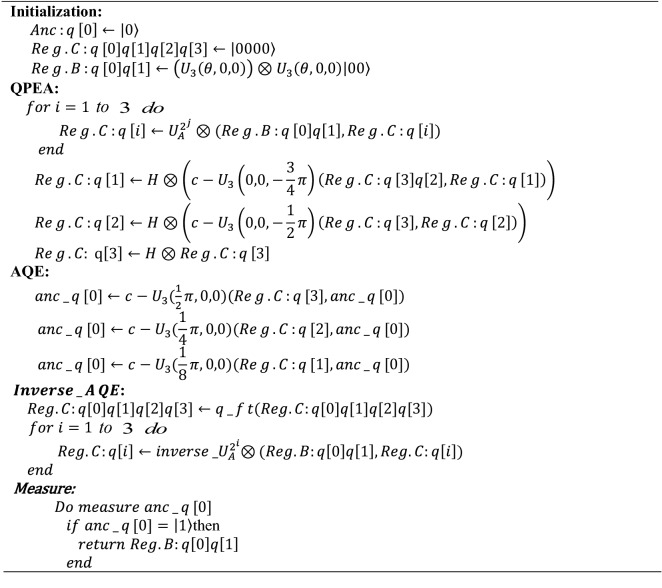

## Data Availability

The datasets used and/or analysed during the current study available from the corresponding author on reasonable request.
